# TUDCA-Treated Mesenchymal Stem Cells Protect against ER Stress in the Hippocampus of a Murine Chronic Kidney Disease Model

**DOI:** 10.3390/ijms20030613

**Published:** 2019-01-31

**Authors:** Jun Hee Lee, Yeo Min Yoon, Sang Hun Lee

**Affiliations:** 1 Department of Pharmacology and Toxicology, University of Alabama at Birmingham School of Medicine, Birmingham, AL 35294, USA; j-school@hanmail.net; 2 Medical Science Research Institute, Soonchunhyang University Seoul Hospital, Seoul 336-745, Korea; yoonboo15@naver.com; 3 Departments of Biochemistry, Soonchunhyang University College of Medicine, Cheonan 330-930, Korea

**Keywords:** chronic kidney disease, mesenchymal stem cells, endoplasmic reticulum stress, tauroursodeoxycholic acid, anti-oxidant

## Abstract

Chronic kidney disease (CKD) leads to the loss of kidney function, as well as the dysfunction of several other organs due to the release of uremic toxins into the system. In a murine CKD model, reactive oxygen species (ROS) generation and endoplasmic reticulum (ER) stress are increased in the hippocampus. Mesenchymal stem cells (MSCs) are one of the candidates for cell-based therapy for CKD; however severe pathophysiological conditions can decrease their therapeutic potential. To address these issues, we established tauroursodeoxycholic acid (TUDCA)-treated MSCs using MSCs isolated from patients with CKD (CKD-hMSCs) and assessed the survival and ROS generation of neural cell line SH-SY5Y cells by co-culturing with TUDCA-treated CKD-hMSCs. In the presence of the uremic toxin *P*-cresol, the death of SH-SY5Y cells was induced by ROS-mediated ER stress. Co-culture with TUDCA-treated CKD-hMSCs increased anti-oxidant enzyme activities in SH-SY5Y cells through the upregulation of the cellular prion protein (PrP^C^) expression. Upregulated PrP^C^ expression in SH-SY5Y cells protected against CKD-mediated ER stress and apoptosis. In an adenine-induced murine CKD model, injection with TUDCA-treated CKD-hMSCs suppressed ROS generation and ER stress in the hippocampus. These results indicate that TUDCA-treated CKD-hMSCs prevent the CKD-mediated cell death of SH-SY5Y cells by inhibiting ER stress. Our study suggests that treatment with TUDCA could be a powerful strategy for developing autologous MSC-based therapeutics for patients with CKD, and that PrP^C^ might be a pivotal target for protecting neural cells from CKD-mediated ER stress.

## 1. Introduction

Chronic kidney disease (CKD) is a major global public health issue, causing several complications such as hypertension, atherosclerosis, aging, and diabetes [1]. In particular, kidney dysfunction leads to the excretion of uremic toxins, which accumulate in the blood and have toxic effects on several types of tissue [2]. Studies using murine CKD models have shown that CKD-mediated oxidative stress is induced in certain regions of the brain, including the cerebral cortex and hippocampus, resulting in cognitive dysfunction [3,4]. *P*-cresol, one of the major uremic toxins, is a protein-bound solute that induces cell apoptosis and senescence [5]. Recent studies have revealed that *P*-cresol induces cellular senescence, inhibition of proliferation, and dysfunction of mitochondria in mesenchymal stem cells (MSCs) [6,7,8]. Therefore, uremic toxins such as *P*-cresol are a major hurdle for stem cell-based therapies, and the development of uremic toxin-resistant stem cells to treat CKD patients is urgently needed.

MSCs are a major source of stem cells for regenerative medicine due to their capacity for self-renewal and multipotency [9]. Although several studies have revealed that the main mechanism of MSC-mediated tissue repair is via their potential for self-renewal and multi-differentiation, their paracrine effect has also been proposed to be an important mechanism of regeneration [10]. In response to injury, MSCs secrete several types of cytokine and chemokine (immunomodulatory, cytoprotective, angiogenic, arteriogenic, antiapoptotic, antioxidant, cell migratory, and homing factors), as well as microvesicles and exosomes [10]. Despite their unique characteristics, the severe pathophysiological conditions of several diseases restrict the beneficial effects of MSCs in regenerative medicine. 

Cellular prion protein (PrP^C^) is a glycosylphosphatidylinositol-anchored glycoprotein whose expression is related to PrP^SC^, the protease resistant misfolded prion isoform of PrP^C^ [11]. Previous reports suggest that PrP^C^ is involved in cell survival, proliferation, and signal transduction [7,12,13,14]. In addition, PrP^C^ increases antioxidant activity under conditions of oxidative stress, leading to inhibition of reactive oxygen species (ROS) generation [7,15,16]. Therefore, PrP^C^ is an interesting molecular target for treating CKD-related cognitive dysfunction induced by oxidative stress. Our previous studies have shown that tauroursodeoxycholic acid (TUDCA), a bile acid, protects against oxidative stress-induced apoptosis in MSCs through the upregulation of PrP^C^ [7,17]. In addition, TUDCA-treated stem cells and progenitor cells have a therapeutic effect in ischemic diseases under conditions of oxidative stress [7,17,18]. These findings suggest that TUDCA-treated stem/progenitor cell-based therapeutics may be one candidate for regenerative medicine for several diseases associated with oxidative stress. This study and other recent studies have found that CKD induces ROS generation and endoplasmic reticulum (ER) stress in the hippocampus [19,20,21]. To reveal the effect of TUDCA-treated CKD-MSCs against *P*-cresol-induced ROS stress in neural cell line SH-SY5Y cells, we co-cultured SH-SY5Y cells with TUDCA-treated CKD-MSCs. We showed that TUDCA-treated MSCs prevent neural cell death induced by CKD-associated ER stress through the upregulation of PrP^C^, highlighting the potential of this pathway for CKD cell-based therapy.

## 2. Results

### 2.1. CKD Increases Neuronal Cell Death via Induction of ER Stress

**Figure 1:**
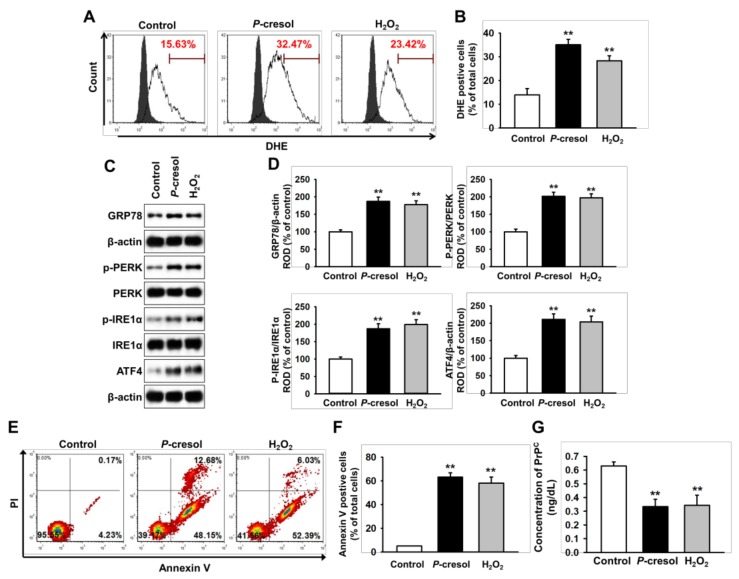
Uremic toxin-induced apoptosis in SH-SY5Y cells through induction of reactive oxygen species (ROS)-mediated endoplasmic reticulum (ER) stress. (**A**) Flow cytometry analysis for dihydroethidium (DHE) in SH-SY5Y cells after treatment with *P*-cresol (500 μM) for 48 h and H_2_O_2_ (200 μM) for 4 h (n = 5). The filled and clear histograms represent the cells in the absence and presence of DHE, respectively. (**B**) Quantification of the percentage of DHE positive cells. (**C**) Western blot analysis for GRP78, phosphorylation of protein kinase R (PKR)-like endoplasmic reticulum kinase (p-PERK), PERK, phosphorylation of inositol-requiring enzyme 1 α (p-IRE1α), IRE1α, and activating transcription factor 4 (ATF4) in SH-SY5Y cells after treatment with *P*-cresol and H_2_O_2_ (*n* = 3). (**D**) The protein levels of (**C**) were determined by densitometry relative to β-actin. (**E**) Flow cytometry analysis for PI/Annexin staining in SH-SY5Y cells after treatment with *P*-cresol and H_2_O_2_ (*n* = 5). (**F**) Quantification of the percentage of Annexin V positive cells. (**G**) The concentration of PrP^C^ in SH-SY5Y cells after treatment with *P*-cresol and H_2_O_2_, as assessed by ELISA (n = 5). Statistical analysis: Values represent the mean ± standard error of the mean (SEM). (**B**) ** *p* < 0.01 vs. control. (**D**) ** *p* < 0.01 vs. control. (**F**) ** *p* < 0.01 vs. control.

To confirm whether CKD induces ROS-mediated neural cell death through ER stress, we assessed cell survival of the neural cell line SH-SY5Y cells following treatment with the uremic toxin *P*-cresol (500 μM) for 48 h. In SH-SY5Y cells, production of ROS (induced H_2_O_2_ (200 μM) for 4 h) was significantly increased following treatment with *P*-cresol, compared to the control (Figure 1A and 1B). In addition, treatment with *P*-cresol significantly increased the levels of ER stress-associated proteins, such as GRP78, phosphorylation of protein kinase R (PKR)-like endoplasmic reticulum kinase (p-PERK), phosphorylation of inositol-requiring enzyme 1 α (p-IRE1α), and activating transcription factor 4 (ATF4) (Figure 1C,D). Annexin/PI staining indicated that *P*-cresol caused a significant increase in apoptosis of SH-SY5Y cells (Figure 1E,F). Interestingly, treatment of SH-SY5Y cells with *P*-cresol and H_2_O_2_ significantly decreased the level of PrP^C^, which has an anti-oxidant effect (Figure 1G). These findings indicate that uremic toxin-mediated ROS induce neural cell death through activation of ER stress, and that PrP^C^ is involved in CKD-mediated neural cell death.

### 2.2. Tudca-Stimulated CKD-hMSCS Protect SH-SY5Y Cells against Uremic Toxin-Induced Oxidative Stress

**Figure 2:**
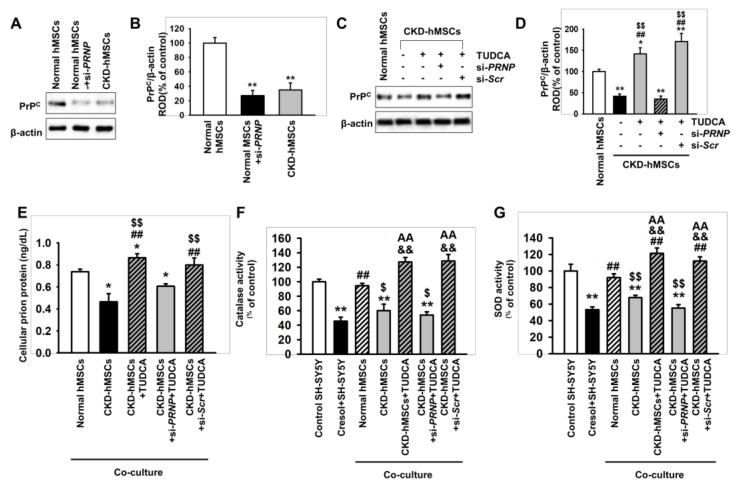
Co-culture of SH-SY5Y cells with TUDCA-stimulated Chronic kidney disease (CKD-hMSCs) increases the activity of anti-oxidant enzymes via upregulation of PrP^C^. (**A**) Western blot showing the expression of cellular prion protein (PrP^C^) in normal human MSCs (hMSCs), normal hMSCs pretreated with *PRNP* (PRioN Protein) siRNA (si-*PRNP*) for 48 h, and hMSCs isolated from a CKD patient (CKD-hMSCs) (*n* = 3). (**B**) The level of PrP^C^ in (**A**) was determined by densitometry relative to β-actin. (**C**) Western blot showing the expression of PrP^C^ in CKD-hMSCs pretreated with TUDCA (1 μM) for 24 h. CKD-hMSCs were pretreated with *PRNP* siRNA (si-*PRNP*) or Scramble siRNA (si-*SCR*) for 48 h, then treated with TUDCA (*n* = 3). (**D**) The expression of PrP^C^ was determined by densitometry relative to β-actin. (**E**) The concentration of PrP^C^ in SH-SY5Y cells after co-culture with hMSCs (*n* = 5). (F and G) Catalase (**F**) and SOD activity (**G**) in SH-SY5Y cells following co-culture with hMSCs. Statistical analysis: Values represent the mean ± SEM. (**B**) ** *p* < 0.01 vs. normal hMSCs. (**D**) ** *p* < 0.01 vs. normal hMSCs, ^##^ *p* < 0.01 vs. CKD-hMSCs, ^$$^ *p* < 0.01 vs. TUDCA-treated CKD-hMSCs pretreated with si-*PRNP*. (**F**) *p < 0.05 vs. normal MSCs, ^##^ *p* < 0.01 vs. CKD-hMSCs, ^$$^ *p* < 0.01 vs. CKD-hMSCs + si-*PRNP* + TUDCA. (**F** and **G**) ** *p* < 0.01 vs. control SH-SY5Y cells without co-culture, ^##^ *p* < 0.01 vs. *P*-cresol+SH-SY5Y without co-culture, ^$^ *p* < 0.05, ^$$^ *p* < 0.01 vs. co-culture with normal hMSCs, ^&&^ *p* < 0.01 vs. co-culture with CKD-hMSCs, ^AA^ *p* < 0.01 vs. co-culture with CKD-hMSCs + si-*PRNP* + TUDCA.

A previous study has shown that PrP^C^ is a key molecule for protecting against oxidative stress in MSCs [7,17]. In addition, our previous study revealed that TUDCA protects MSCs against ER stress caused by oxidative stress through the regulation of PrP^C^ [7], showing that the secretion of PrPC was significantly decreased after treatment of SH-SY5Y cells with *P*-cresol and H_2_O_2_. Therefore, to investigate whether TUDCA-stimulated CKD-derived MSCs protect neural cells against uremic toxin-induced oxidative stress, we performed co-culture of SH-SY5Y cells with human MSCs isolated from CKD patients (CKD-hMSCs) with the addition of *P*-cresol, and confirmed the anti-oxidant effect on SH-SY5Y cells. First, we assessed the expression level of PrP^C^, which was significantly decreased in CKD-hMSCs, compared to normal MSCs (Figure 2A,B). However, treatment of CKD-hMSCs with TUDCA (1 μM) for 24 h significantly increased the expression of PrP^C^, compared with non-treated CKD-hMSCs and normal MSCs (Figure 2C,D). Furthermore, in a co-culture assay, the secretion of PrP^C^ by CKD-hMSCs was significantly decreased in the presence of SH-SY5Y cells compared to that in cells co-cultured with normal MSCs (Figure 2E). However, TUDCA-treated CKD-hMSCs significantly enhanced the secretion of PrP^C^ from SH-SY5Y cells in co-culture (Figure 2E). In addition, catalase and superoxide dismutase (SOD) activities were significantly increased in SH-SY5Y cells co-cultured with TUDCA-treated CKD-hMSCs with the presence of uremic toxin, compared with SH-SY5Y cells alone or those co-cultured with untreated CKD-hMSCs (Figure 2F,G). Furthermore, silencing PrP^C^, which is encoded by the human PRioN Protein (*PRNP*) gene, blocked these anti-oxidant effects in SH-SY5Y cells (Figure 2F,G). These data indicate that co-culture of SH-SY5Y cells with TUDCA-treated CKD-hMSCs protects against uremic toxin-induced oxidative stress through the activation of anti-oxidant enzymes induced by PrP^C^ expression.

### 2.3. TUDCA-Treated CKD-hMSCs Suppress Uremic Toxin-Induced ER Stress in SH-SY5Y Cells via Upregulation of PrP^C^

**Figure 3:**
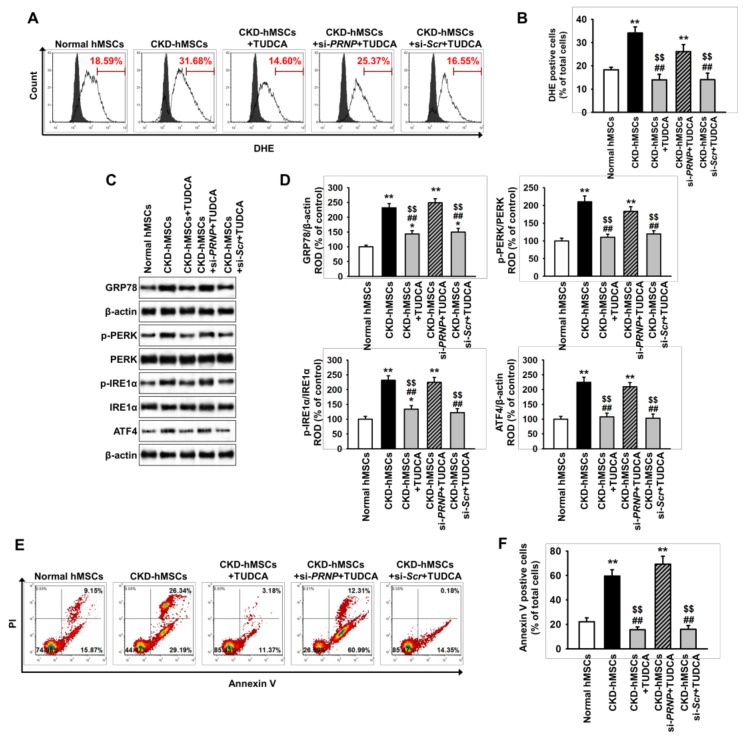
Co-culture with TUDCA-treated CKD-hMSCs prevents apoptosis of SH-SY5Y cells in the presence of uremic toxin through protection of ER stress. In the presence of *P*-cresol, SH-SY5Y cells were co-cultured with hMSCs and subsequently analyzed by flow cytometry analysis and western blot. (**A**) Flow cytometry analysis for DHE staining in SH-SY5Y cells after co-culture with hMSCs (*n* = 5). The filled and clear histograms represent cells in the absence and presence of DHE, respectively. (**B**) Quantification of the percentage of DHE positive cells from (**A**). (**C**) Western blot analysis for GRP78, p-PERK, PERK, p-IRE1α, IRE1α, and ATF4 in SH-SY5Y cells after co-culture with hMSCs (*n* = 3). (**D**) The protein levels of (**C**) were determined by densitometry relative to β-actin. (**E**) Flow cytometry analysis following PI/Annexin V staining of SH-SY5Y cells co-cultured with hMSCs (*n* = 5). (**F**) Quantification of the percentage of Annexin V positive cells from (**E**). Statistical analysis: Values represent the mean ± SEM. (**B**) ** *p* < 0.01 vs. co-culture with normal hMSCs, ^##^ *p* < 0.01 vs. co-culture with CKD-hMSCs, ^$$^ *p* < 0.01 vs. co-culture with CKD-hMSCs + si-*PRNP* + TUDCA. (**D**) * *p* < 0.05, ** *p* < 0.01 vs. co-culture with normal hMSCs, ^##^ *p* < 0.01 vs. co-culture with CKD-hMSCs, ^$$^ *p* < 0.01 vs. co-culture with CKD-hMSCs + si-*PRNP* + TUDCA. (**F**) ** *p* < 0.01 vs. co-culture with normal hMSCs, ^##^ *p* < 0.01 vs. co-culture with CKD-hMSCs, ^$$^ *p* < 0.01 vs. co-culture with CKD-hMSCs + si-*PRNP* + TUDCA.

To explore whether TUDCA-treated CKD-hMSCs protect against neural cell death induced by uremic toxin-mediated ER stress, we investigated the ER stress-mediated signaling pathway and SH-SY5Y cell death in the presence of *P*-cresol after co-culture with TUDCA-treated CKD-hMSCs. Production of ROS was higher in SH-SY5Y co-cultured with CKD-hMSCs than in those co-cultured with normal hMSCs (Figure 3A,B). However, treatment of CKD-hMSCs with TUDCA significantly decreased the levels of ROS in SH-SY5Y, compared with those co-cultured with untreated CKD-hMSCs (Figure 3A,B). This protective effect of TUDCA-treated CKD-hMSCs on ROS production in SH-SY5Y cells was blocked by PrP^C^ knockdown (Figure 3A,B). Under uremic toxin conditions, activation of the ER stress-mediated signaling pathway, including GRP78, p-PERK, p-IRE1α, and ATF4, was significantly increased in SH-SY5Y cells co-cultured with CKD-hMSCs, compared with those co-cultured with normal hMSCs (Figure 3C,D). In contrast, co-culture with TUDCA-treated CKD-hMSCs significantly inhibited activation of the ER stress-mediated proteins in a manner dependent on PrP^C^ expression (Figure 3C,D). Furthermore, a flow cytometry based Annexin V/ propidium iodide (PI) assay revealed that uremic toxin-induced apoptosis of SH-SY5Y cells was significantly reduced by co-culture with TUDCA-treated CKD-hMSCs, and this effect was reversed by PrP^C^ knockdown (Figure 3E,F). These results suggest that TUDCA-treated CKD-hMSCs prevent ER stress-induced neural cell death induced by uremic toxin in a manner dependent on PrP^C^.

### 2.4. TUDCA-Treated CKD-hMSCs Prevent ROS-Mediated ER Stress in The Hippocampus of CKD Mice through Prp^c^ Expression

**Figure 4:**
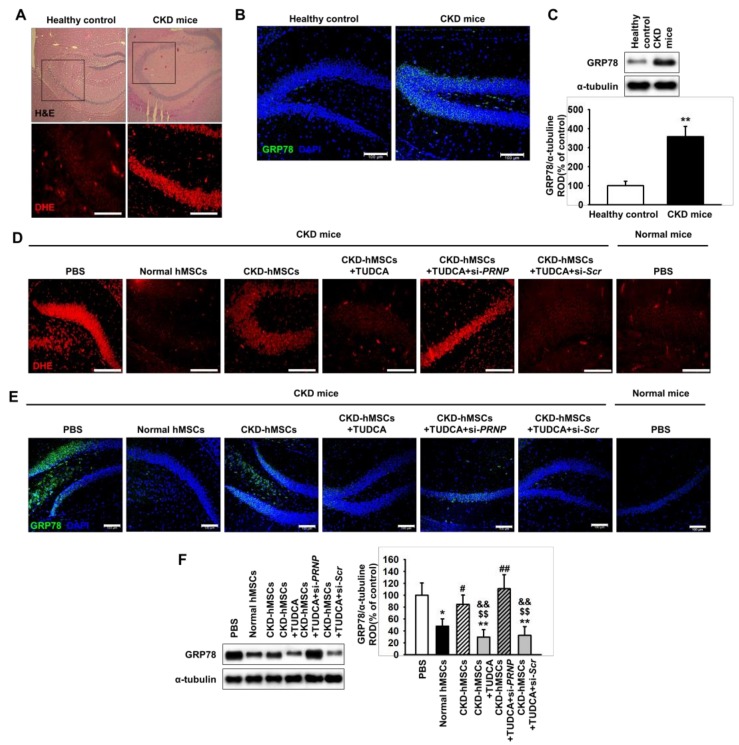
Co-culture of SH-SY5Y cells with TUDCA-stimulated CKD-hMSCs increases the activity of anti-oxidant enzymes via upregulation of PrP^C^. (**A**) In healthy mice (*n* = 3) or murine CKD model (*n* = 3), hematoxylin and eosin (H and E; upper images) staining and dihydroethidium (DHE, red; cropped images) staining were performed on hippocampus sections. (**B**) Immunofluorescence staining for glucose-regulated protein 78 (GRP78) in the hippocampus of a CKD mouse (*n* = 3). Scale bars = 100 μm. (**C**) Western blot analysis for GRP78 in the hippocampus of a healthy mouse and a CKD mouse (*n* = 3). The protein levels were determined by densitometry relative to α-tubulin. (**D**) DHE staining (red) the hippocampus of a murine CKD model was injected with either phosphate buffer saline (PBS) or each type of hMSCs. For each mouse (*n* = 3), the hippocampus was isolated and analyzed 25 days after the first injection. (**E**) Immunofluorescence staining for GRP78 in the hippocampus of a murine CKD model was injected with either PBS or each type of hMSCs (*n* = 3). Scale bar =100 μm. (**F**) Western blot analysis for GRP78 in the hippocampus of a murine CKD model (*n* = 3). The protein levels were determined by densitometry relative to α-tubulin. Statistical analysis: Values represent the mean ± SEM. (C) ** *p* < 0.01 vs. healthy control. (**F**) * *p* < 0.05, ** *p* < 0.01 vs. PBS, ^#^ *p* < 0.05, ^##^ *p* < 0.01 vs. normal hMSCs, ^$$^ *p* < 0.01 vs. CKD-hMSCs, ^&&^ *p* < 0.01 vs. CKD-hMSCs + TUDCA + si-*PRNP*.

To investigate whether CKD induces the neural production of ROS, dihydroethidium (DHE) staining was used to measure the level of ROS in the brain of a CKD mouse. In the hippocampus, the level of ROS was significantly increased in CKD mice compared with healthy control mice (Figure 4A). To further explore whether ER stress is associated with CKD-induced hippocampal ROS production, we measured the expression of the ER stress marker glucose-regulated protein 78 (GRP78) in the brain of a CKD mouse. Western blot analysis and immunofluorescence staining for GRP78 in the hippocampus showed that the expression of GRP78 in the CKD mouse was significantly higher than that in the healthy control mouse (Figure 4B,C). These results indicate that CKD induces the production of ROS in the hippocampus through ER stress.

To further assess the protective effect of TUDCA-treated CKD-hMSCs on CKD-induced ER stress in neural cells in vivo, we employed the CKD mouse model. We injected normal hMSCs, CKD-hMSCs, TUDCA-treated CKD-hMSCs, TUDCA-treated CKD-hMSCs with si-*PRNP*, and TUDCA-treated CKD-hMSCs with scramble siRNA intravenously via the tail on day 0, 5, and 10. On day 25 after the first injection, ROS production and expression of the ER stress marker GRP78, were assessed by immunohistochemistry on the hippocampus of CKD mice. This revealed that ROS levels were higher in the phosphate buffer saline (PBS) injection group than the normal hMSC group, whereas the levels of ROS were significantly increased by injection with CKD-hMSCs, compared with normal hMSCs (Figure 4D). However, the TUDCA-treated CKD-hMSC group displayed a significant decrease in ROS levels, and this reduction was dependent on expression of PrP^C^ (Figure 4D). In addition, western blot analysis and immunofluorescence staining for GRP78 in the hippocampus revealed that ER stress was significantly inhibited by injection with TUDCA-treated CKD-hMSCs and this effect was also reversed by silencing of PrP^C^ (Figure 4E,F). These results indicate that TUDCA-treated CKD-hMSCs protect against the production of ROS and ER stress in the hippocampus of CKD mice. 

## 3. Discussion

In this study, we provide evidence that the generation of ROS is induced in the hippocampus of an adenine-induced murine model, resulting in ROS-mediated ER stress and cell death in neural cell line SH-SY5Y cells. In addition, we have shown that co-culture of SH-SY5Y cells with TUDCA-treated CKD-hMSCs inhibits uremic toxin-induced ER stress and cell death in SH-SY5Y cells through the upregulation of PrP^C^. In particular, in a murine CKD model, injection with TUDCA-treated CKD-hMSCs suppressed ROS production and ER stress, suggesting that TUDCA-treated hMSCs could prevent oxidative stress in the hippocampus of those suffering with CKD. The paracrine effect is one possible mechanism by which TUDCA-treated CKD-hMSCs protect SH-SY5Y cells against cell death due to *P*-cresol-induced oxidative stress. In *P*-cresol-treated MSCs, TUDCA increases the secretion of IL-10 [7], which inhibits free radical generation [22]. PrP^C^ has been detected in the ER and Golgi, suggesting that it could be packaged into secretory vesicles [23,24]. Our results have shown that in a co-culture assay, TUDCA-treated CKD-hMSCs increased the level of PrP^C^ in SH-SY5Y cells, suggesting that PrP^C^ might be delivered from TUDCA-treated CKD-hMSCs to SH-SY5Y cells by secretory vesicles. However, further studies are needed to determine the precise mechanisms by which cytokines/growth factors are secreted in MSCs in response to TUDCA treatment and how PrP^C^ is delivered from TUDCA-treated MSCs to SH-SY5Y cells. 

TUDCA is one of the bile acids which has been used for the treatment of several liver diseases, such as primary sclerosing cholangiolitis and biliary cirrhosis [25]. In neurodegenerative diseases, TUDCA also protects neural cell apoptosis and neuronal loss by upregulating cell survival pathways [26]. Furthermore, recent studies indicate that TUDCA-stimulated stem/progenitor cells enhance their functionalities and therapeutic effects in ischemic diseases [7,17,18]. TUDCA-stimulated endothelial progenitor cells (EPCs) increase angiogenesis in a murine hind limb ischemia model by enhancing recruitment of EPCs to ischemic injured tissues [18]. In addition, TUDCA rescues cellular senescence in aged EPCs through reduction of cyclin-dependent kinase inhibitor 1A (p21) and ROS levels, with the result that TUDCA-treated senescent EPCs enhance blood vessel regeneration in a hind limb model [18]. Under ischemia-mediated ER stress conditions, TUDCA inhibits ER stress-induced apoptosis in MSCs through the RAC (Rho family of GTPases)—alpha serine/threonine-protein kinase (Akt)-PrP^C^ signaling pathway [17]. In particular, TUDCA-treated MSCs increase functional recovery in a murine hind limb ischemia model by enhancing the activity of the anti-oxidative enzyme, manganese-dependent superoxide dismutase (MnSOD) [17]. Our recent study has shown that TUDCA exerts a protective effect against oxidative stress in MSCs under the presence of uremic toxin via the upregulation of PrP^C^ [7]. This study indicates that treatment with TUDCA significantly increases the expression of PrP^C^ in CKD-hMSCs, compared with that in non-treated CKD-hMSCs. PrP^C^ plays a role in several intracellular signal transduction pathways and is also released to other cells during cell–cell interaction by exosomes [27]. Our results have shown that co-culture of SH-SY5Y cells with TUDCA-treated CKD-hMSCs increases the PrP^C^ level in SH-SY5Y cells, with the result that *P*-cresol-induced ER stress and apoptosis in SH-SY5Y cells are inhibited by increased anti-oxidant enzyme activities. These findings suggest that TUDCA-stimulated CKD-MSCs could protect against neural cell death induced by uremic toxin-induced oxidative stress through the secretion of PrP^C^. However, further investigation is needed to elucidate the precise mechanism of TUDCA-mediated secretion of PrP^C^ in MSCs.

PrP^C^ is a glycoprotein which attaches to the cell membrane [28]. Abnormal conversion of PrP^C^ to PrP^Sc^ generates prions, leading to neurodegenerative diseases [29]. Although the abnormal form, PrP^Sc^, causes dysfunction of the nervous system, PrP^C^ is associated with various cellular and physiological processes, such as neural differentiation, protection of oxidative stress, inhibition of neurodegeneration, and self-renewal of stem cells [30,31,32]. Previous studies have revealed that *P*-cresol increases the production of ROS in MSCs [17]. In particular, oxidative stress is induced by an imbalance in ROS and increases as CKD progresses [33], indicating that ROS generation is closely related to the stage of CKD. In MSCs, the upregulation of PrP^C^ protects against apoptosis caused by ischemia-induced oxidative stress [12]. PrP^C^ also regulates the expression of MnSOD, resulting in protection of MSCs against ROS-mediated ER stress in an Akt-dependent manner [17]. In addition, PrP^C^ prevents apoptosis of MSCs under conditions of uremic toxin-mediated ER stress [7]. Furthermore, PrP^C^ contributes to long-term neuroprotection in the ischemic brain and PrP^C^ deficiency causes sensitivity to oxidative stress in brain ischemia [34,35,36]. Our results indicate that co-culture of SH-SY5Y cells with TUDCA-treated CKD-hMSCs increases PrP^C^ expression in SH-SY5Y cells, resulting in induction of catalase and SOD activity. Augmentation of anti-oxidant enzyme activities results in a reduction of ROS and inhibition of ER stress, leading to protection against apoptosis in SH-SY5Y cells under the presence of uremic toxins. These results indicate that the augmentation of PrP^C^ levels by co-culture with TUDCA-treated CKD-hMSCs protects SH-SY5Y cells against uremic toxin-induced ER stress through the activation of anti-oxidant enzymes, suggesting that PrP^C^ plays a key role in protection against ER stress in neural cells under conditions of CKD-mediated oxidative stress. Moreover, these findings suggest that TUDCA might be a powerful priming agent for enhancing the antioxidant effect of MSCs in patients with CKD.

**Figure 5:**
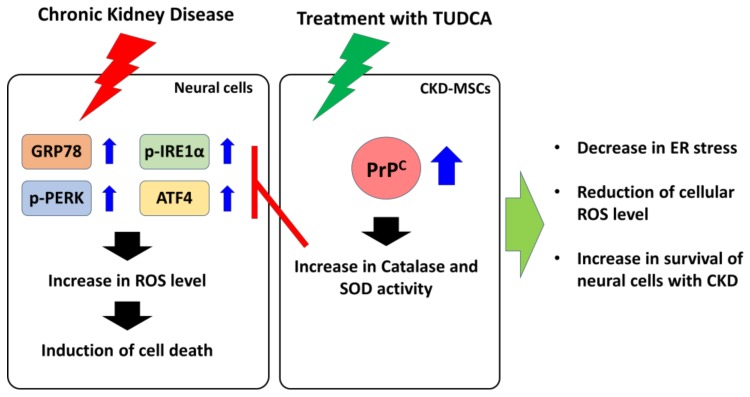
Schematic representation of the proposed mechanisms by which TUDCA-treated CKD-MSCs protect against neural cell apoptosis induced by CKD-mediated ER stress. TUDCA-treated CKD-MSCs induce the expression and secretion of PrP^C^, leading to increased levels of PrP^C^ in neural cells. Under the presence of uremic toxin, the upregulation of PrP^C^ inhibits CKD-mediated ER stress in neural cells through the induction of catalase and SOD activities, resulting in increased survival of neural cells in subjects with CKD. Blue array means that increase expression or activation of protein. Red “T” arrow means that block expression or activation of protein. Black array means cell signal pathway. Green arrow means TUDCA-treated CKD-MSCs finally protection pathway.

Although MSCs are one of the powerful sources for autologous stem cell-based therapeutics, pathophysiological conditions in several chronic diseases decrease their therapeutic efficacy. Our findings indicate that TUDCA-treated CKD-hMSCs protect against CKD-mediated ROS generation and ER stress in the hippocampus of a murine CKD model in a PrP^C^-dependent manner. These results suggest that treatment with TUDCA might be an effective strategy for the development of autologous MSC-based therapeutics in patients with CKD. However, since there are several murine CKD models which are induced by various etiologies (genetic, autoimmune, infectious, environmental, dietary, and medication), each with differing characteristics, they cannot exactly simulate and predict responses in patients with CKD [37]. Therefore, further studies using other CKD models are needed to reveal the full protective effects of TUDCA-treated CKD-hMSCs against ROS generation in the hippocampus. Taken together, our study indicates that TUDCA-treated CKD-MSCs protect against neural cell death induced by ROS-associated ER stress in CKD through the PrP^C^-catalase/SOD axis (Figure 5). These findings suggest that TUDCA-treated MSCs could be a strong candidate for autologous stem cell-based therapeutics for CKD, and that PrP^C^ might be a key molecule for protection against ROS-associated ER stress in the hippocampus of CKD patients.

## 4. Materials and Methods

### 4.1. Cell Culture of Human MSCs 

Human adipose tissue-derived normal MSCs (hMSCs) and MSCs isolated from CKD patients (CKD-hMSCs) were obtained from Soonchunhyang University Seoul Hospital (IRB: SCHUH 2015-11-017, 2 November 2015). CKD-hMSCs were derived from the adipose tissue of CKD patients, according to the estimated glomerular filteration rate [eGFR] < 35 mL/(min⋅1.73 m^2^) for more than 3 months (stage 3b). The supplier certified that CD44 and Sca-1 were expressed on the surface of hMSCs. Normal hMSCs and CKD-hMSCs were cultured in α-Minimum Essential Medium (Gibco BRL, Gaithersburg, MD, USA) supplemented with 10% (*v*/*v*) fetal bovine serum (FBS; Gibco BRL) and 100 U/mL penicillin/streptomycin (Gibco BRL). Both cell types were grown in a humidified 5% CO_2_ incubator at 37 °C.

### 4.2. Culture of SH-SY5Y Cells

The SH-SY5Y cell line was purchased from Seoul University Hospital (Seoul, Korea) and cultured in DMEM (Gibco BRL) supplemented with 10% (*v*/*v*) FBS (Gibco BRL) in a humidified 5% CO_2_ incubator at 37 °C. To establish CKD conditions, SH-SY5Y cells were washed twice with PBS and placed in fresh DMEM with *P*-cresol (500 μM) for 48 h. 

### 4.3. Co-Culture of SH-SY5Y Cells with hMSCs

SH-SY5Y cells and hMSCs were co-cultured in Millicell Cell Culture Plates (Millipore, Billerica, MA, USA) in a humidified 5% CO_2_ incubator at 37 °C. SH-SY5Y cells were seeded in the lower compartments, followed by exposure to *P*-cresol for 24 h. MSCs were then seeded onto the transwell membrane inserts and the plates were incubated for a further 24 h, following which the SH-SY5Y cells were assessed by several assays.

### 4.4. Western Blot Assay

Protein extracts were separated via 8% to 12% sodium dodecyl sulfate-polyacrylamide gel electrophoresis followed by transfer to nitrocellulose membrane. After blocking membranes with 5% skim milk for 1 h, glucose-regulated protein 78 (GRP78) primary antibodies against PrP^C^, protein kinase-like endoplasmic reticulum kinase (PERK), phospho-PERK (p-PERK), inositol-requiring enzyme 1α (IRE1α), p-IRE1α, activating transcription factor 4 (ATF4), and β-actin (Santa Cruz Biotechnology, Dallas, TX, USA) were incubated overnight. After washing with PBS, the membranes were incubated with goat anti-rabbit immunoglobulin g (IgG) or goat anti-mouse IgG conjugated to horseradish peroxidase (Santa Cruz Biotechnology). The bands were detected by enhanced chemiluminescence (Amersham Pharmacia Biotech, England, UK).

### 4.5. Silencing of PrP^C^ Expression by RNA Interference

CKD-hMSCs (2 × 10^5^) seeded in 60-mm dishes were transfected with siRNA in serum-free Opti-MEM (Gibco BRL) using Lipofectamine 2000 (Thermo Fisher Scientific, Waltham, MA, USA) according to the manufacturer’s instructions. At 48 h post-transfection, total protein was extracted, and gene expression was determined through western blot analysis. The siRNA used to target PRNP and a scrambled sequence were synthesized by Bioneer (Daejeon, Korea).

### 4.6. Flow Cytometric Analysis

Cell apoptosis was assessed using a Cyflow Cube 8 (Partec, Münster, Germany) after staining the cells with propidium iodide (PI) and Annexin V-fluorescein isothiocyanate (FITC). Data analysis was performed using standard FSC Express (De Novo Software, Los Angeles, CA, USA).

### 4.7. Immunohistochemistry

Mouse brain tissue was fixed with 4% paraformaldehyde (Sigma-Aldrich, St. Louis, MS, USA) and embedded in paraffin. Immunofluorescence staining was performed using primary antibodies against GRP78 (Santa Cruz Biotechnology) and secondary antibodies Alexa-488 (Thermo Fisher Scientific). Nuclei were stained with 4′,6-diaminido-2-phenylindol (DAPI; Sigma-Aldrich), and immunostained samples were observed using confocal microscopy (Olympus, Tokyo, Japan).

### 4.8. DHE Staining

DHE (Sigma-Aldrich) was used to measure superoxide anions in CKD mouse brain sections. Each group of sections was exposed to DHE (10 μM) for 30 min at 37 °C. After washing with PBS three times, samples were visualized by confocal microscopy (Olympus) at 488 nm excitation and 590 nm emission.

### 4.9. Catalase activity

Cells were seeded in 100-mm tissue culture plates and grown to 70% to 75% confluence. After washing twice in PBS, cells were resuspended in lysis buffer (1% Triton X-100 in 50 mM Tris-HCl [pH 7.4] containing 150 mM NaCl, 5 mM EDTA, 2 mM Na_3_VO_4_, 2.5 mM Na_4_PO_7_, 100 mM NaF, and protease inhibitors). Samples were incubated for 30 min on ice and were centrifuged at 14000 rpm for 30 min at 4 °C. After measuring the protein concentration of the supernatant fraction, catalase activity was measured using a Catalase Assay Kit (Sigma-Aldrich) according to the manufacturer’s instructions. 

### 4.10. Superoxide Dismutase Activity

Cells were harvested, and protein was isolated using extraction buffer. Cell lysates (50 µg) were treated with SOD, and the signal was immediately measured each minute for 15 min using an ELISA reader (BMG labtech, Ortenberg, Germany) at 450 nm.

### 4.11. Ethics Statement 

All animal care procedures and experiments were approved by the Institutional Animal Care and Use Committee of Soonchunhyang University Seoul Hospital (IACUC2013-5, 16 February 2014) and were performed in accordance with the National Research Council (NRC) Guidelines for the Care and Use of Laboratory Animals. The experiments were performed on 8-week-old male BALB/c nude mice (Biogenomics, Seoul, Korea) maintained on a 12 h light/dark cycle at 25 °C in accordance with the regulations of Soonchunhyang University Seoul Hospital.

### 4.12. The CKD Model

Eight-week-old male BALB/c nude mice were fed an adenine-containing diet (0.25% adenine) for 1 to 2 weeks. Mouse body weight was measured every week. The mice were randomly assigned to 1 of 4 groups containing 10 mice each. After euthanasia, blood was stored at −80 °C to measure blood urea nitrogen (control: 15.8939 ± 1.2360 mg/dL, CKD: 71.8677 ± 2.1999 mg/dL) and creatinine (control: 0.3657 ± 0.0264 mg/dL, CKD: 1.9483 ± 0.1573 mg/dL). In addition, enlarged kidneys were observed.

### 4.13. Statistical Analysis

Results are expressed as the mean ± SEM. All of the experiments were analyzed by one-way analysis of variance (ANOVA). Some comparisons of ≥3 groups were made using the Bonferroni-Dunn test. A *p* value < 0.05 was considered statistically significant.

## References

[1] Peired A.J., Sisti A., Romagnani P. (2016). Mesenchymal Stem Cell-Based Therapy for Kidney Disease: A Review of Clinical Evidence. Stem Cells Int..

[2] Vanholder R., De Smet R., Glorieux G., Argiles A., Baurmeister U., Brunet P., Clark W., Cohen G., De Deyn P.P., Deppisch R. (2003). Review on uremic toxins: Classification, concentration, and interindividual variability. Kidney Int..

[3] Deng G., Vaziri N.D., Jabbari B., Ni Z., Yan X.X. (2001). Increased tyrosine nitration of the brain in chronic renal insufficiency: Reversal by antioxidant therapy and angiotensin-converting enzyme inhibition. J. Am. Soc. Nephrol..

[4] Fujisaki K., Tsuruya K., Yamato M., Toyonaga J., Noguchi H., Nakano T., Taniguchi M., Tokumoto M., Hirakata H., Kitazono T. (2014). Cerebral oxidative stress induces spatial working memory dysfunction in uremic mice: Neuroprotective effect of tempol. Nephrol. Dial. Transpl..

[5] Azevedo M.L., Bonan N.B., Dias G., Brehm F., Steiner T.M., Souza W.M., Stinghen A.E., Barreto F.C., Elifio-Esposito S., Pecoits-Filho R. (2016). p-Cresyl sulfate affects the oxidative burst, phagocytosis process, and antigen presentation of monocyte-derived macrophages. Toxicol. Lett..

[6] Lee J.H., Yun C.W., Hur J., Lee S.H. (2018). Fucoidan Rescues p-Cresol-Induced Cellular Senescence in Mesenchymal Stem Cells via FAK-Akt-TWIST Axis. Mar. Drugs.

[7] Yun S.P., Yoon Y.M., Lee J.H., Kook M., Han Y.S., Jung S.K., Lee S.H. (2018). Tauroursodeoxycholic Acid Protects against the Effects of P-Cresol-Induced Reactive Oxygen Species via the Expression of Cellular Prion Protein. Int. J. Mol. Sci..

[8] Yoon Y.M., Han Y.S., Yun C.W., Lee J.H., Kim R., Lee S.H. (2018). Pioglitazone Protects Mesenchymal Stem Cells against P-Cresol-Induced Mitochondrial Dysfunction via Up-Regulation of PINK-1. Int. J. Mol. Sci..

[9] Lv F.J., Tuan R.S., Cheung K.M., Leung V.Y. (2014). Concise review: The surface markers and identity of human mesenchymal stem cells. Stem Cells.

[10] Liang X., Ding Y., Zhang Y., Tse H.F., Lian Q. (2014). Paracrine mechanisms of mesenchymal stem cell-based therapy: Current status and perspectives. Cell Transpl..

[11] Westergard L., Christensen H.M., Harris D.A. (2007). The cellular prion protein (PrP(C)): Its physiological function and role in disease. Biochim. Biophys. Acta.

[12] Lee J.H., Han Y.S., Lee S.H. (2017). Potentiation of biological effects of mesenchymal stem cells in ischemic conditions by melatonin via upregulation of cellular prion protein expression. J. Pineal Res..

[13] Lee J.H., Han Y.S., Yoon Y.M., Yun C.W., Yun S.P., Kim S.M., Kwon H.Y., Jeong D., Baek M.J., Lee H.J. (2017). Role of HSPA1L as a cellular prion protein stabilizer in tumor progression via HIF-1alpha/GP78 axis. Oncogene.

[14] Lee J.H., Yun C.W., Han Y.S., Kim S., Jeong D., Kwon H.Y., Kim H., Baek M.J., Lee S.H. (2018). Melatonin and 5-fluorouracil co-suppress colon cancer stem cells by regulating cellular prion protein-Oct4 axis. J. Pineal Res..

[15] Han Y.S., Yun S.P., Lee J.H., Kwon S.H., Kim S., Hur J., Lee S.H. (2018). C-Met-Activated Mesenchymal Stem Cells Rescue Ischemic Damage via Interaction with Cellular Prion Protein. Cell. Physiol. Biochem..

[16] Zanetti F., Carpi A., Menabo R., Giorgio M., Schulz R., Valen G., Baysa A., Massimino M.L., Sorgato M.C., Bertoli A. (2014). The cellular prion protein counteracts cardiac oxidative stress. Cardiovasc. Res..

[17] Yoon Y.M., Lee J.H., Yun S.P., Han Y.S., Yun C.W., Lee H.J., Noh H., Lee S.J., Han H.J., Lee S.H. (2016). Tauroursodeoxycholic acid reduces ER stress by regulating of Akt-dependent cellular prion protein. Sci. Rep..

[18] Cho J.G., Lee J.H., Hong S.H., Lee H.N., Kim C.M., Kim S.Y., Yoon K.J., Oh B.J., Kim J.H., Jung S.Y. (2015). Tauroursodeoxycholic acid, a bile acid, promotes blood vessel repair by recruiting vasculogenic progenitor cells. Stem Cells.

[19] Impellizzeri D., Esposito E., Attley J., Cuzzocrea S. (2014). Targeting inflammation: New therapeutic approaches in chronic kidney disease (CKD). Pharmacol. Res..

[20] Inagi R. (2009). Endoplasmic reticulum stress in the kidney as a novel mediator of kidney injury. Nephron Exp. Nephrol..

[21] Kosuge Y., Osada N., Shimomura A., Miyagishi H., Wada T., Ishige K., Shimba S., Ito Y. (2018). Relevance of the hippocampal endoplasmic reticulum stress response in a mouse model of chronic kidney disease. Neurosci. Lett..

[22] Dokka S., Shi X., Leonard S., Wang L., Castranova V., Rojanasakul Y. (2001). Interleukin-10-mediated inhibition of free radical generation in macrophages. Am. J. Physiol. Lung Cell. Mol. Physiol..

[23] Cardinale A., Filesi I., Vetrugno V., Pocchiari M., Sy M.S., Biocca S. (2005). Trapping prion protein in the endoplasmic reticulum impairs PrPC maturation and prevents PrPSc accumulation. J. Boil. Chem..

[24] Stewart R.S., Harris D.A. (2005). A transmembrane form of the prion protein is localized in the Golgi apparatus of neurons. J. Boil. Chem..

[25] Vang S., Longley K., Steer C.J., Low W.C. (2014). The Unexpected Uses of Urso- and Tauroursodeoxycholic Acid in the Treatment of Non-liver Diseases. Glob. Adv. Health Med..

[26] Gronbeck K.R., Rodrigues C.M., Mahmoudi J., Bershad E.M., Ling G., Bachour S.P., Divani A.A. (2016). Application of Tauroursodeoxycholic Acid for Treatment of Neurological and Non-neurological Diseases: Is There a Potential for Treating Traumatic Brain Injury?. Neurocrit. Care.

[27] Robertson C., Booth S.A., Beniac D.R., Coulthart M.B., Booth T.F., McNicol A. (2006). Cellular prion protein is released on exosomes from activated platelets. Blood.

[28] Stahl N., Borchelt D.R., Hsiao K., Prusiner S.B. (1987). Scrapie prion protein contains a phosphatidylinositol glycolipid. Cell.

[29] Prusiner S.B., Groth D.F., Bolton D.C., Kent S.B., Hood L.E. (1984). Purification and structural studies of a major scrapie prion protein. Cell.

[30] Black S.A., Stys P.K., Zamponi G.W., Tsutsui S. (2014). Cellular prion protein and NMDA receptor modulation: Protecting against excitotoxicity. Front. Cell Dev. Biol.

[31] Shi F., Yang Y., Wang T., Kouadir M., Zhao D., Hu S. (2016). Cellular Prion Protein Promotes Neuronal Differentiation of Adipose-Derived Stem Cells by Upregulating miRNA-124. J. Mol. Neurosci..

[32] Lopes M.H., Santos T.G. (2012). Prion potency in stem cells biology. Prion.

[33] Dounousi E., Papavasiliou E., Makedou A., Ioannou K., Katopodis K.P., Tselepis A., Siamopoulos K.C., Tsakiris D. (2006). Oxidative stress is progressively enhanced with advancing stages of CKD. Am. J. Kidney Dis..

[34] Shyu W.C., Lin S.Z., Chiang M.F., Ding D.C., Li K.W., Chen S.F., Yang H.I., Li H. (2005). Overexpression of PrPC by adenovirus-mediated gene targeting reduces ischemic injury in a stroke rat model. J. Neurosci..

[35] Brown D.R., Nicholas R.S., Canevari L. (2002). Lack of prion protein expression results in a neuronal phenotype sensitive to stress. J. Neurosci. Res..

[36] Weise J., Sandau R., Schwarting S., Crome O., Wrede A., Schulz-Schaeffer W., Zerr I., Bahr M. (2006). Deletion of cellular prion protein results in reduced Akt activation, enhanced postischemic caspase-3 activation, and exacerbation of ischemic brain injury. Stroke.

[37] Yang H.C., Zuo Y., Fogo A.B. (2010). Models of chronic kidney disease. Drug Discov. Today Dis. Models.

